# Incidental Discovery of a Left Ventricular Aneurysm After a Syncopal Episode

**DOI:** 10.7759/cureus.17979

**Published:** 2021-09-14

**Authors:** Christopher E Roberts, Hunaid N Rana, Brian Wood, Zeiad Hussain

**Affiliations:** 1 Medicine, University of South Alabama, Mobile, USA; 2 Radiology, University of South Alabama, Mobile, USA; 3 Interventional Radiology, University of South Alabama, Mobile, USA

**Keywords:** myocardial infarction complication, ventricular aneurysm thrombus, chest radiograph, computed tomography, coronary artery angiography, left ventricular aneurysm

## Abstract

A left ventricular aneurysm is a rare post myocardial infarction complication. Ventricular aneurysms form as post-ischemic cardiac remodeling creates a weaker, fibrotic area that may bulge outwards against interventricular pressures over time. Patients with ventricular aneurysms have increased mortality and are at higher risk of various cardiac complications, such as cardiac arrest, arrhythmias, thrombus formation, reduced cardiac output, or aneurysmal rupture. Prompt diagnosis and treatment are critically important in these patients. We highlight the hospital course of a patient with an extensive cardiac history presenting for syncope with the discovery of a left ventricular aneurysm. The radiographic features of the left ventricular aneurysm are described, as well as formation, risk factors, and complications.

## Introduction

A left ventricular aneurysm (LVA) is an uncommon post-myocardial infarction (MI) complication that forms in 10-35% of patients [1-3]. However, LVA incidence has been trending down likely due to improvements in MI treatment [4]. An LVA is formed as the scar tissue created after the ischemic event is weaker than surrounding cardiac tissue, allowing for bulging out of the scarred area. While small LVAs may be asymptomatic, large LVAs are associated with many potentially life-threatening complications [5]. These high-risk patients need a multidisciplinary team-based approach with both medical and surgical management.

## Case presentation

A 59-year-old Caucasian male with an extensive cardiac history presented to the emergency department for intermittent pre-syncopal episodes over the past three to four weeks and a syncopal episode immediately prior to arrival. His past medical history includes coronary artery disease (CAD), ischemic cardiomyopathy with a reduced ejection fraction of less than 20% due to prior anterior myocardial infarction at 35 years of age, atrial fibrillation, history of multiple GI bleeds, and hyperlipidemia. The patient described pre-syncopal episodes as feeling “dizzy” and unrelated to exertion, body position (standing, sitting, lying down, or different head/neck positions), medication usage, or timing throughout the day. Prior to the syncopal episode, the patient was walking in his house whereupon he felt dizzy, sat in a chair, lost consciousness, and fell to the floor. The patient was found by his son and taken to the emergency department. The patient denied chest pain or neurological deficits.

**Figure 1:**
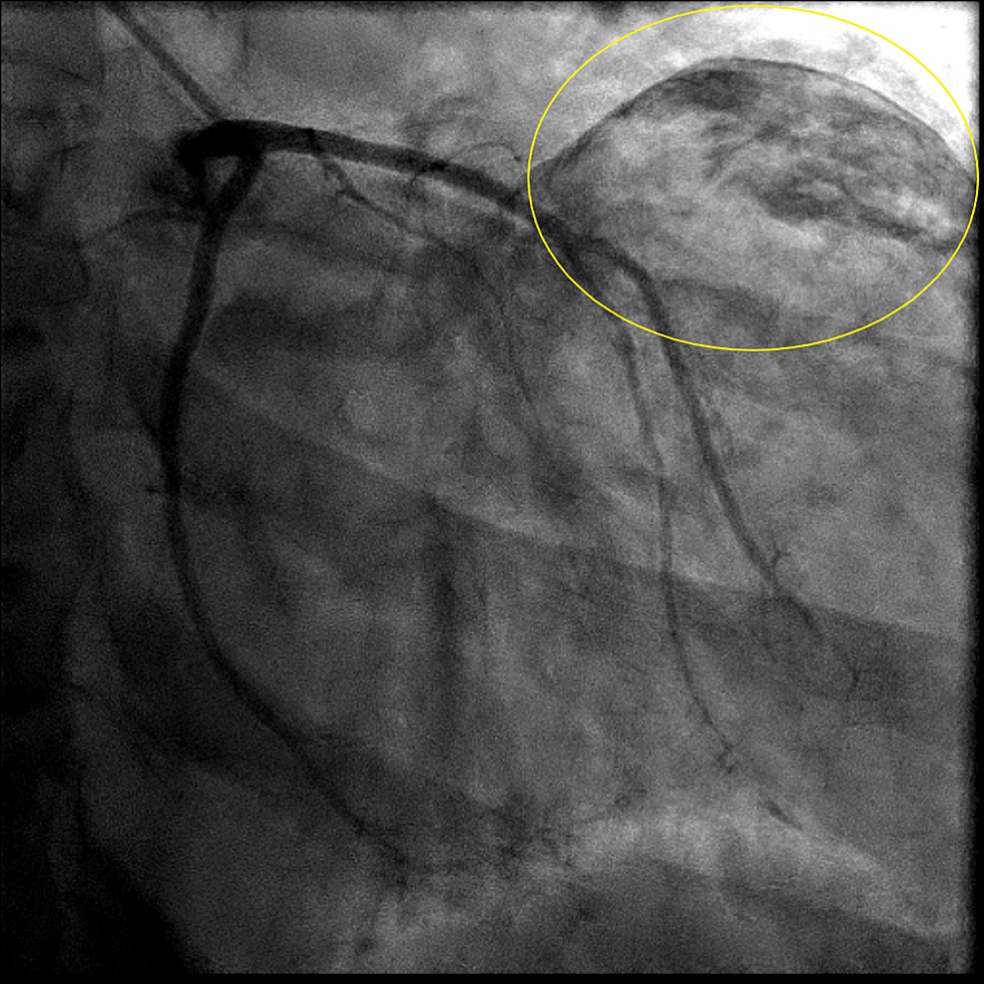
Anteroposterior view of coronary angiography of the left coronary artery with a calcified mass seen projecting over the left heart (yellow circle).

**Figure 2:**
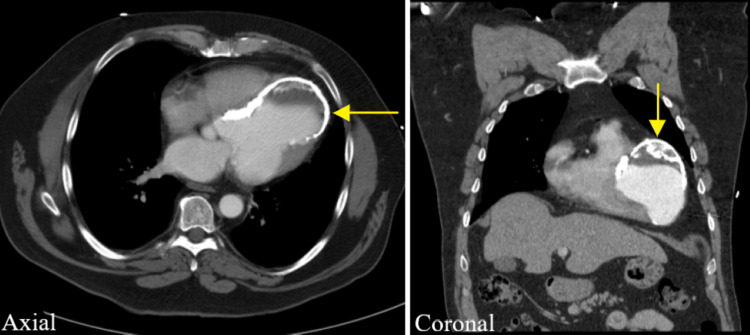
Axial and coronal intravenous contrasted CT images showing the calcified border of left ventricular aneurysm (yellow arrows). A partially calcified thrombus is also seen within the aneurysm.

**Figure 3:**
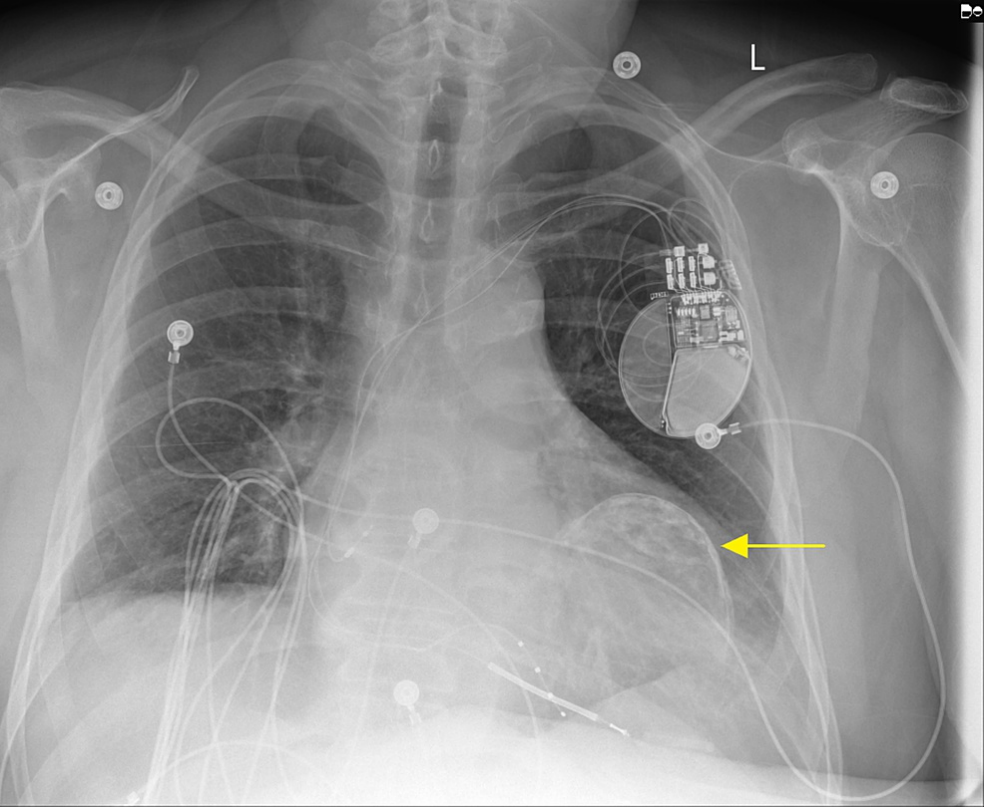
Posteroanterior chest radiograph showing placement of ICD with the calcified ventricular aneurysm wall seen projecting over the left heart (yellow arrow).

At the emergency department, a CT scan of the head and cervical spine were obtained, which did not reveal any acute hemorrhage or fracture. Serial troponin and creatine kinase (CK) levels were not elevated and an ECG showed bradycardia and left bundle branch block. Due to the patient’s cardiac history, the decision to admit to the hospital for coronary angiography and implantable cardioverter defibrillator (ICD) placement was made. During angiography, a left-sided calcified mass projecting over the left ventricle was seen (Figure 1). A CT chest with contrast was obtained, which demonstrated an LVA with a calcified border and partially calcified thrombus (Figure 2). The thrombus was thought to be chronic due to the calcification and anticoagulation was ultimately deferred due to chronicity as well as the patient’s history of major GI bleeding. After the discovery of the LVA, the decision for ICD placement was maintained. Following ICD placement, a chest radiograph was obtained, which showed device placement (Figure 3).

The patient was discharged the following day. At a two-week cardiology clinic follow-up, the patient did not report any syncopal episodes since ICD placement. Seven months post ICD placement, the patient noticed swelling, erythema, and purulent drainage around the pacemaker pocket site. The patient was diagnosed with a pacemaker pocket infection and scheduled for ICD explant and reimplantation, as well as starting antibiotic therapy. After ICD re-implantation and infection resolution, the patient has been seen multiple times in the cardiology clinic reporting symptom improvement post ICD placement and no syncopal episodes.

## Discussion

LVAs often form from an anterior MI, in which ischemia causes tissue necrosis leading to an inflammatory cascade. The inflammatory phase lasts one to five days post MI and is characterized initially by neutrophil influx followed by macrophage migration. These acute inflammatory cells degrade necrotic tissue and signal for recruitment of other inflammatory cells. Next, the proliferative phase begins, which may last for weeks. This phase is characterized by cardiac fibroblasts and myofibroblasts beginning to synthesize and deposit extracellular matrix proteins, such as collagen and adhesive molecules. Finally, the maturation phase begins and lasts months, in which the extracellular matrix continues development and attains peak tensile strength [6]. As the scar never regains the initial tensile strength as healthy myocardium, the pressure within the heart can cause dilation of the scar over time and aneurysm formation. Ventricular rupture is an acute life-threatening complication of an LVA. Aneurysms within the early regenerative phases have an increased risk of rupture as the tissue is relatively thin and weak. However, in late phases, as scar tissue is deposited and remodeled, the risk of rupture may decrease [7]. As LVAs are associated with a wide variety of cardiac complications, accurate diagnosis is of high importance.

An ECG can aid the diagnosis of LVA in patients presenting with cardiac complaints. ECG findings of LVA include persistent ST segment elevation, Q waves, and flattened T segments. However, ECG findings of LVA can be difficult to differentiate from acute MI. Criteria based upon LVA having smaller T waves than acute MI have been suggested to help differentiate between the two diagnoses. Klein et al. suggest acute MI is favored if the sum of T-wave amplitudes in leads V1-V4 divided by the sum of QRS amplitudes in leads V1-V4 is greater than 0.22 or if any single lead (V1-V4) has a T-wave amplitude to QRS amplitude ratio greater than or equal to 0.36 [8]. However, these ECG criteria are not definitive and should be considered along with the rest of the clinical picture. In patients presenting for an acute MI, CK and cardiac troponin I may be elevated, but peak levels of these enzymes are not predictive of LVA formation. However, peak N-terminal pro b-type natriuretic peptide (NT-pro BNP) levels may be significantly higher in MI patients who later develop LVA [9]. Imaging may be needed to help differentiate LVA from other causes of cardiac complaints.

Diagnosis of an LVA may be made through a wide variety of imaging modalities. Radiography can show the dilation of the aneurysm causing enlargement of the heart border. If present, calcifications may also be seen within the aneurysmal wall. However, radiography is relatively nonspecific, thus further imaging may be used to help confirm the diagnosis. Echocardiography and ventriculography can show dilation of the aneurysm within the ventricle and as well as characterization of contractility or motion abnormalities within the aneurysmal segment [10]. CT and MRI would additionally be able to characterize the aneurysm as well as better estimate three-dimensional volume. Furthermore, three-dimensional imaging can help differentiate a true LVA from other differentials diagnoses, such as pseudoaneurysm or diverticulum [11,12]. Correct diagnosis of LVA from other differentials is essential to begin appropriate treatment and reduce potential complications.

An LVA are associated with an increased risk of cardiac complications due to altered electrical conductivity, impaired myocardial contraction, and abnormal blood flow within the diseased heart. Patients with LVAs may have increased rates of ventricular arrhythmia, cardiac arrest, reduced cardiac output, left ventricular thrombus formation, and ischemic stroke compared to patients without LVA [3,5]. Patients with LVA additionally have an increased risk of aneurysmal rupture, especially in early aneurysm formation phases [7]. LVA is also associated with worse outcome in acute MI compared to absence of LVA. Patients with LVA had significantly higher mortality, increased rates of cardiac and noncardiac (such as stroke or acute kidney injury) complications, and greater usage of hospital resources compared to patients without LVA [1,2]. The increased risks and complications of cardiac disease associated with LVA indicate the importance of intensive management. Medical management often involves anticoagulation, reduction in cardiac risk factors, or symptom control. Surgical repair indications include intractable arrhythmia, heart failure, or angina; techniques vary based upon the size and position of the aneurysm [13,14]. Coordination of a multidisciplinary healthcare team can help provide the best care for patients with LVA.

## Conclusions

We highlighted a patient presenting with a syncopal episode and extensive cardiac history, including acute MI, who was found to have an incidental LVA on imaging. We discussed risk factors, formation, and complications associated with LVA. Additionally, imaging modalities used to help diagnose LVA were reviewed.
